# Geriatric chronic recurrent multifocal osteomyelitis (CRMO) mimicking multifocal multiple myeloma: a first in an octogenarian

**DOI:** 10.1007/s00256-024-04653-z

**Published:** 2024-03-19

**Authors:** Jonathan Sgaglione, Andrew Muran, Matthew Rhode, Howard J. Goodman, Morris C. Edelman, Suhail Ahmed Shah, Andrew S. Greenberg, Shachar Kenan

**Affiliations:** 1 Donald and Barbara Zucker School of Medicine, Hofstra/Northwell 500 Hofstra Blvd, Hempstead, NY 11549 USA; 2 Northwell Health, Long Island Jewish Medical Center Department of Orthopaedic Surgery, 270-05 76th Avenue, New York, NY 11040 USA; 3 Pediatric Pathology Division, Northwell Health, Long Island Jewish Medical Center Department of Pathology, 270-05 76th Avenue, New York, NY 11040 USA; 4 Department of Internal Medicine, Northwell Health, Long Island Jewish Medical Center Department of Orthopaedic Surgery, 270-05 76th Avenue, New York, NY 11040 USA; 5 Orthopaedic Associates of Manhasset, 600 Northern Blvd, Lake Success, NY 11021 USA

**Keywords:** CRMO, Chronic recurrent multifocal osteomyelitis, Pathologic fracture, Octogenarian

## Abstract

Chronic recurrent multifocal osteomyelitis (CRMO), an autoinflammatory bone disorder characterized by non-bacterial osteomyelitis causing recurrent multifocal bone lesions, is a well-known, yet uncommon pediatric condition that rarely affects adults; to date, it has never been diagnosed over the age of 75. The following report will discuss the first octogenarian diagnosed with CRMO and therefore represents an exceptionally rare presentation of a rare disease. An 83-year-old woman presented with progressive right shoulder, forearm, and hip pain, with associated weight loss and global weakness, requiring a wheelchair for mobility. Imaging revealed a pathologic right ulna fracture in addition to lytic lesions of the right proximal humerus and proximal femur. The clinical picture was thus that of a patient with probable multiple myeloma versus metastatic disease. After an extensive workup, however, the lesions were not malignant; histologic findings were instead suggestive of chronic osteomyelitis with negative cultures. Given the multifocal nature of this condition, combined with a lack of clinical symptoms of infection, a diagnosis of CRMO was rendered. The patient underwent intramedullary nailing of the right femur and splinting of the ulna, with a subsequent remarkable recovery to painless ambulation, complete union of the right ulna fracture, and resolution of the lytic lesions without receiving any targeted medical treatment. This case highlights the importance of maintaining CRMO on the differential for multifocal skeletal lesions, regardless of age. Performing a thorough workup with necessary imaging, biopsy, and culture are critical to establishing this diagnosis, which can only made as a diagnosis of exclusion.

## Introduction

Chronic recurrent multifocal osteomyelitis (CRMO) is an autoinflammatory bone disorder resulting from imbalanced cytokine expression that causes multifocal bone lesions with frequent recurrence [1, 2]. CRMO is characterized by recurrent bone pain due to inflammation of the metaphyses and diaphyses of long bones and typically occurs in the lower limbs and pelvis [2, 3]. Patients with CRMO often have comorbid autoimmune diseases and a family history of autoimmunity [4]. With a median age of onset of 10 years old, CRMO is typically regarded as a pediatric condition that also occurs more frequently in females [3–5]. CRMO in adults is very rare, with only 10% of patients being over the age of 20. The largest reported study of CRMO patients contained only 31 adult cases out of 486, with an average age of onset of 33 years in the adult cohort [6]. Rarer still are cases of CRMO in patients over the age of 50. There has only been one other published case of CRMO in a patient over the age of 65 [7]. The current case describes the first octogenarian with CRMO reported to date. Our case describes an 83-year-old woman who presented with multifocal skeletal permeative lesions mimicking multiple myeloma. A comprehensive workup was performed ruling out common benign, malignant, infectious, and inflammatory conditions, with the final result of CRMO selected as a diagnosis of exclusion. This case report will review the current literature and management of geriatric CRMO.

## Case report

### History of present illness

An 83-year-old woman with a past medical history of hypertension, hypersensitivity pneumonitis, and atrial fibrillation status post cerebrovascular accident (on Xarelto), presented with two months of progressive right arm pain, acutely worsened after bumping her right elbow 3 weeks prior to presentation. She also reported an associated unintentional thirty-pound weight loss over the past 6 months and required a wheelchair for mobility due to global deconditioning and weakness. She denied any oncologic family history, night sweats, fevers, or dermatologic conditions.

### Imaging

**Fig. 1:**
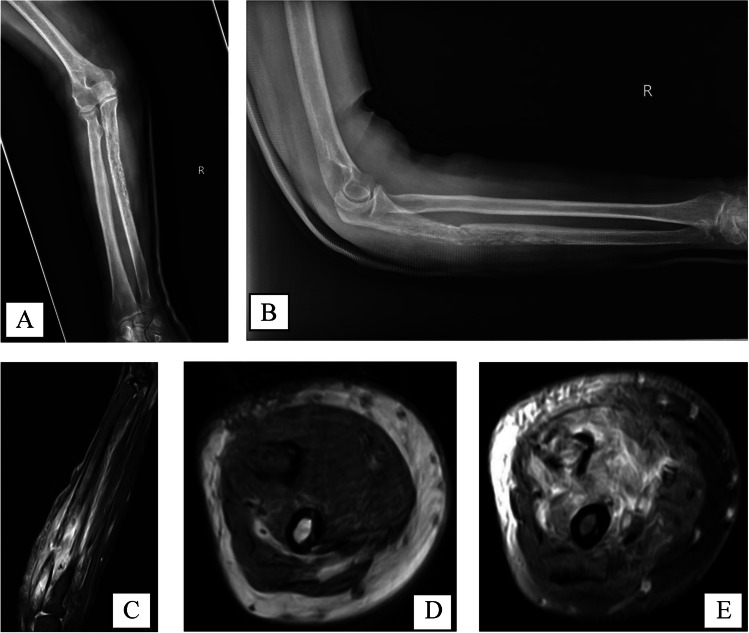
4/2021 — AP (**A**) and lateral (**B**) right forearm radiographs demonstrating an infiltrative lesion along the proximal to mid-ulnar diaphysis with associated minimally displaced angulated pathologic fracture. Coronal FS PD FSE (**C**), axial T1 (**D**), and FS PD FSE (**E**) MRI sequences showing complete destruction of the ulnar cortex with diffuse circumferential fluid hyperintensity in the surrounding soft tissues consistent with surrounding edema

**Fig. 2:**
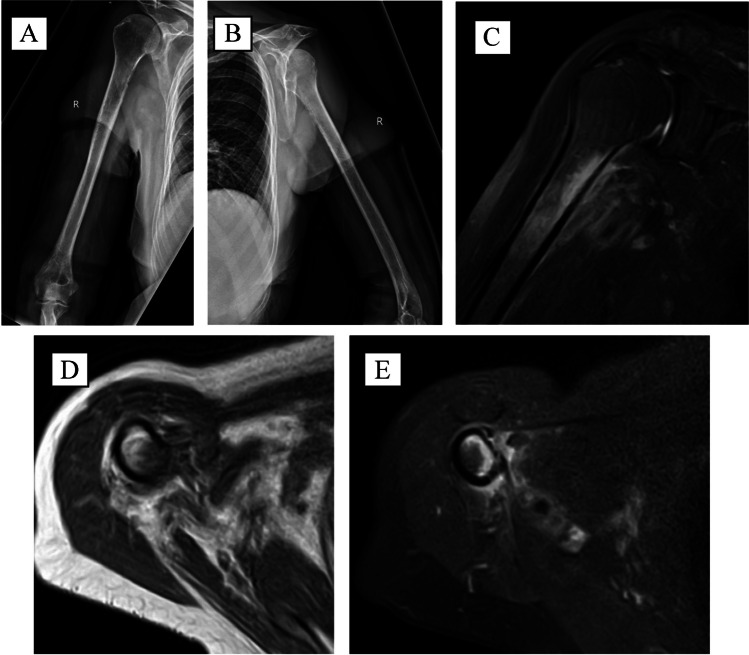
4/2021 — AP (**A**) and lateral (**B**) right humerus radiographs demonstrating an infiltrative lesion involving the proximal diaphysis with medial cortical erosion. Coronal T2 FS FSE (**C**), axial T1 (**D**), and T2 FS FSE (**E**) MRI sequences showing erosion of the medial cortex at the level of the proximal diaphysis with surrounding intramedullary and extramedullary edema

Initial radiological evaluation with radiographs and magnetic resonance imaging (MRI) revealed multiple lytic lesions involving the right proximal humerus and ulna (Figs. 1–2). There was a 6-cm infiltrative permeative lesion within the right proximal humerus with medial cortical destruction and an 8-cm permeative lesion of the ulna with circumferential cortical destruction and pathologic fracture. The radiographic differential diagnosis at this stage included multiple myeloma, metastatic disease, lymphoma, and less likely, osteomyelitis given the multifocal presentation. Rarer conditions on the radiographic differential included Erdheim-Chester and Rosai-Dorfman disease.

**Fig. 3:**
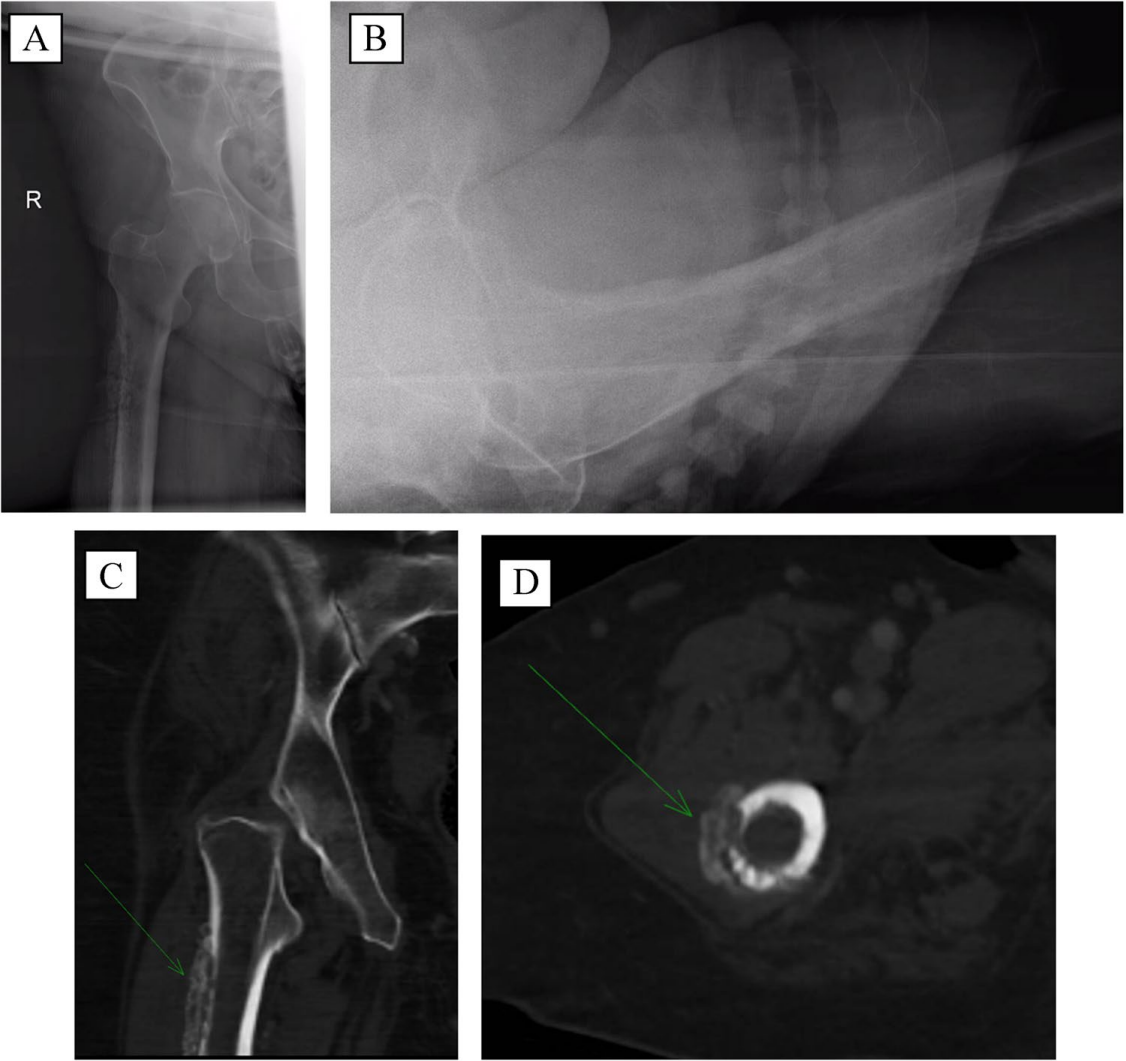
4/2021 — AP (**A**) and lateral (**B**) right hip radiographs demonstrating an infiltrative lesion involving the right proximal femoral diaphysis with complete lateral cortical destruction and adjacent periosteal reaction consistent with impending pathologic fracture. Coronal computed tomography (CT) (**C**) and axial CT (**D**) similarly showing lateral proximal diaphyseal cortical destruction with adjacent exuberant lateral cortical periosteal reaction and osseous proliferation (see arrows)

Metastatic workup including computed tomography (CT) chest, abdomen, and pelvis, and a radiographic skeletal survey revealed an additional large infiltrative lesion of the right proximal femur with associated lateral cortical destruction, consistent with an impending pathologic fracture, otherwise negative for any visceral involvement (Fig. 3). The Gestalt of these imaging findings was most consistent with multiple myeloma. A nuclear bone scan was not performed as it is not a specific test and would not change the acute clinical management of this patient.

### Clinical course

Laboratory results including complete blood count (CBC), comprehensive metabolic panel (CMP), erythromycin sedimentation rate (ESR), C-reactive protein (CRP), lactate dehydrogenase (LDH), serum protein electrophoresis (SPEP), and urine protein electrophoresis (UPEP) were significant for elevated ESR (62 mm/h) and CRP (56.6 mg/L) levels, consistent with an acute phase response, otherwise within normal limits.

**Fig. 4:**
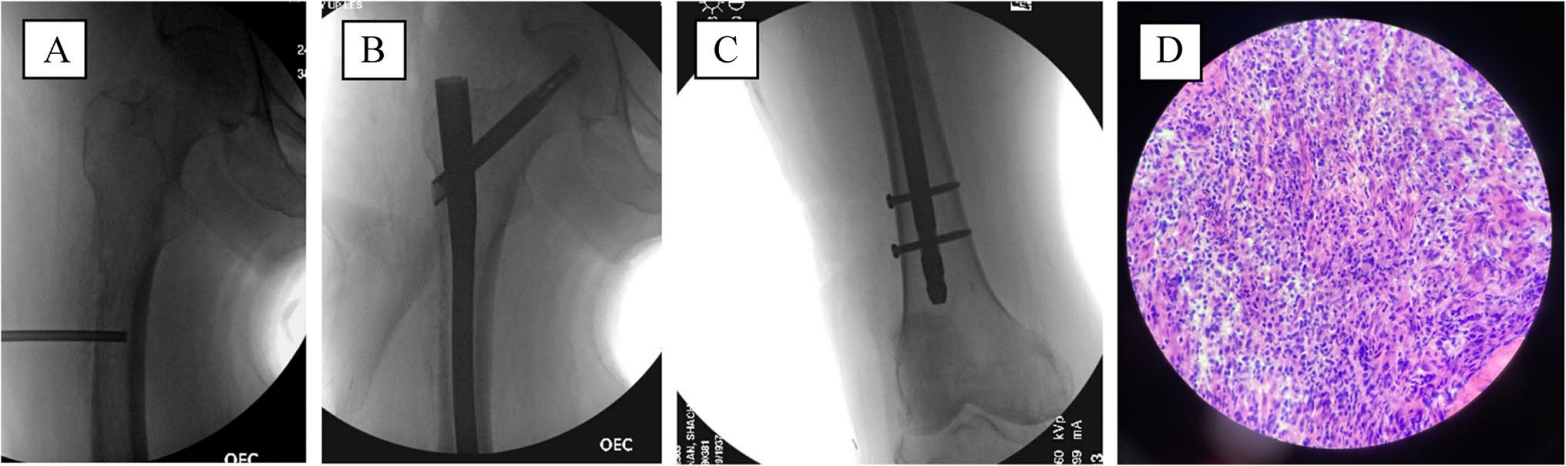
4/27/2021 — Core biopsy under fluoroscopy (**A**) and insertion of long right femoral intramedullary nail (**B**, **C**) after ruling out malignancy on frozen section (**D**)

Subsequent clinical secondary survey of the patient was significant for right hip tenderness to palpation, pain with range of motion, and inability to bear weight — findings which were initially overlooked given the lack of complaints related to her right hip. Given the high risk of fracture, she was indicated for right femur open biopsy, frozen section, prophylactic intramedullary nailing, and right iliac crest bone marrow biopsy and aspiration. The ulna fracture was immobilized with a posterior splint during this time, awaiting final pathology results. The initial frozen section of the right femur revealed hematopoietic cells without any evidence of malignancy; therefore, it was decided to proceed with intramedullary nailing (Fig. 4).

**Fig. 5:**
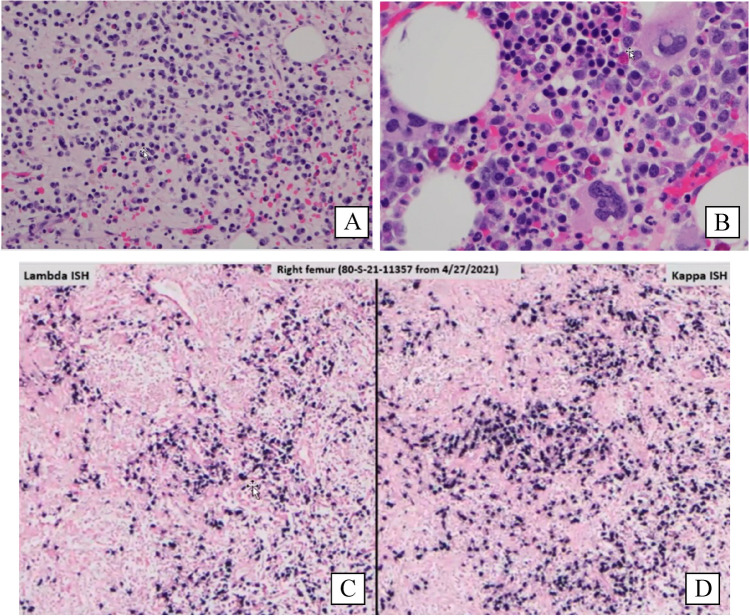
Right femur histology: low (**A**) and high (**B**) power magnification demonstrating a mixed inflammatory cell infiltrate suggestive of chronic osteomyelitis. Mixed lambda (**C**) and Kappa (**D**) in situ hybridization (ISH) rules out multiple myeloma

**Fig. 6:**
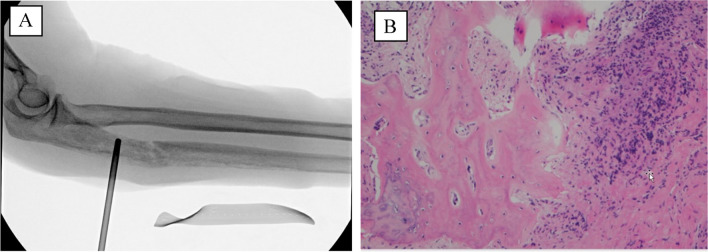
5/12/2021 — Right proximal ulna core biopsy under fluoroscopy (**A**) with histologic appearance similar to the right femur, demonstrating lamellar cortical bone with reactive woven bone formation, medullary fibrosis, and chronic inflammation with scattered plasma cells (**B**)

Final pathology results of the right femur revealed a mixed inflammatory cell infiltrate suggestive of chronic osteomyelitis, although intraoperatively there was no evidence of purulence (Fig. 5). All blood cultures were otherwise negative, and the right iliac crest biopsy and flow cytometry revealed no diagnostic abnormalities. Given the lack of a clear diagnosis, she was indicated for an open biopsy and culture of the right ulna (Fig. 6a).

### Pathology

Histologic examination of the right proximal femur and proximal ulna lesions were nearly identical. Both displayed a mixed chronic inflammatory infiltrate, predominantly lymphoplasmacytic with macrophages, myofibroblasts, and endothelial lined vascular channels suggestive of chronic osteomyelitis (Figs. 5 and 6b). Further stains including AFB and GMS were negative for microorganisms. Immunohistochemical stains for CD3, CD20, CD138, CD163, and LCA revealed LCA+ inflammatory cells including CD138+ plasma cells, CD3+ T cells, rare CD20+ B cells, and scattered CD163+ macrophages. Final cultures, however, were negative for any bacterial or fungal growth. The diagnosis of osteomyelitis from latent syphilis was also ruled out as the rapid plasma reagin (RPR) test was negative. An exhaustive rheumatologic series of tests including HLAB27, ANA, B2G, CCP, C-ANCA, P-ANCA, and RF were all negative. Anticardiolipin and QuantiFERON-TB gold were positive, although both were thought to be false positives, unrelated to the underlying pathology. Cumulatively, these findings were most consistent with CRMO as a diagnosis of exclusion.

### Clinical follow-up

**Fig. 7:**
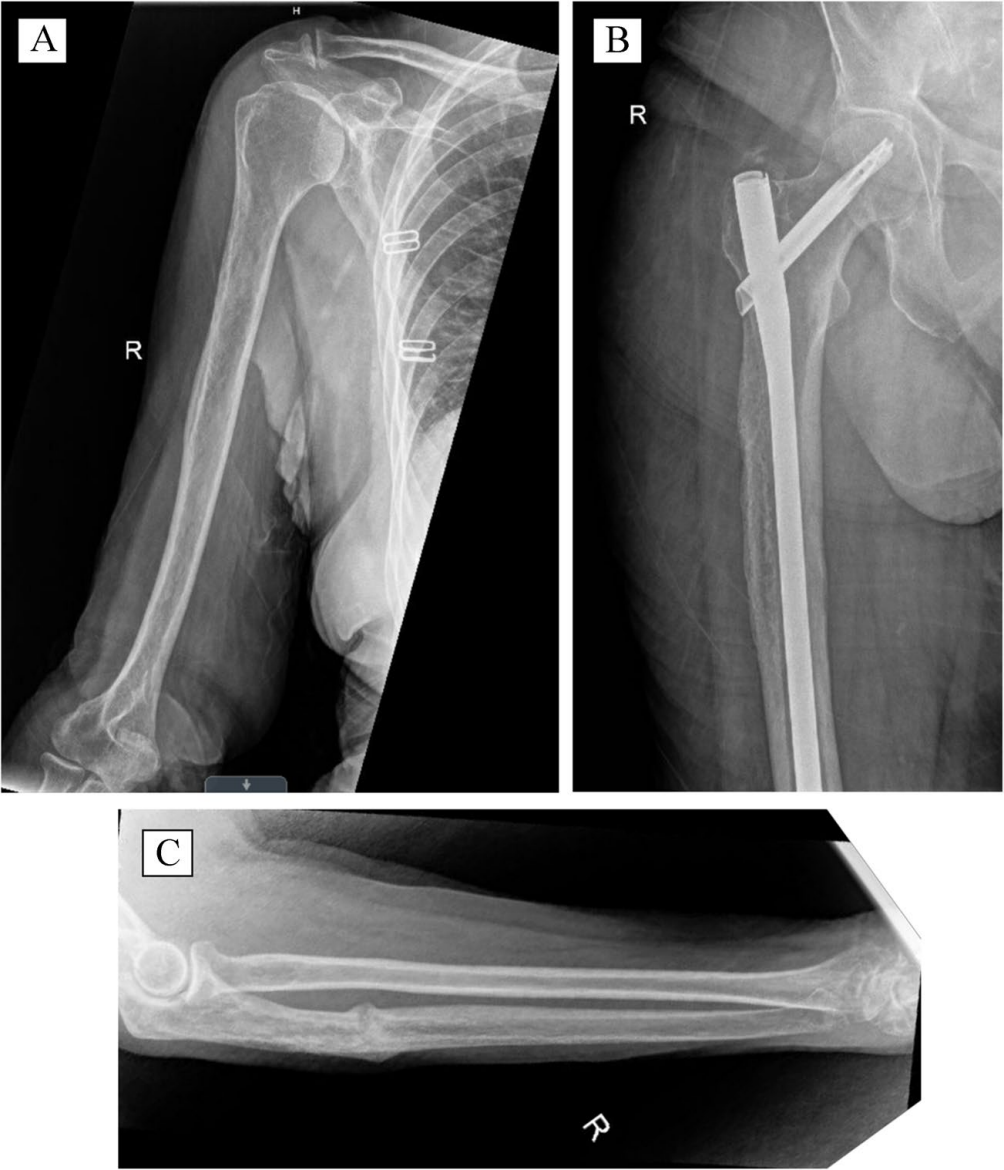
8/10/2021 — Right humerus (**A**), right femur (**B**), and right ulna (**C**) at 14-week postoperative follow-up visit, with interval bridging callus, union, and reossification of all pathologic fractures

**Fig. 8:**
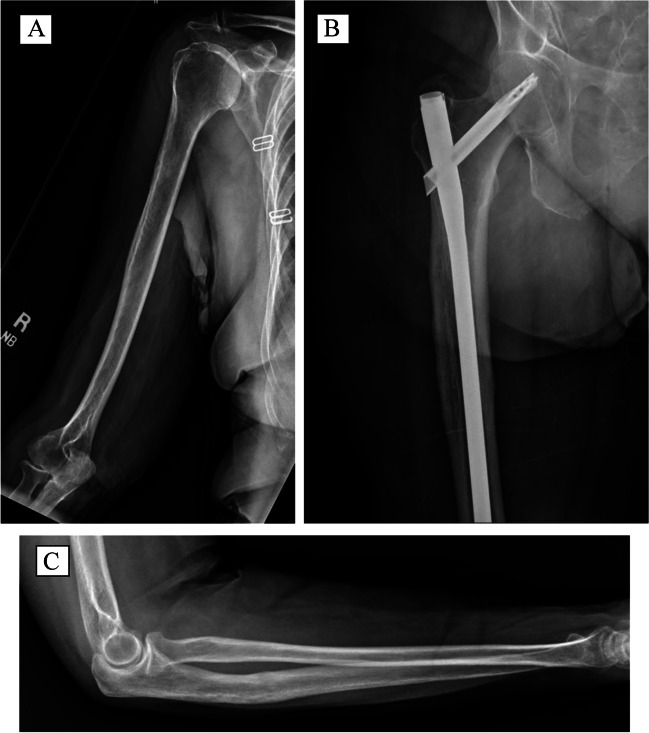
2/17/2022 — Right humerus (**A**), right femur (**B**), and right ulna (**C**) at 9-month postoperative follow-up visit, with complete bone remodeling around the prior pathologic fractures, with no evidence of local recurrence

Postoperatively, the patient was made weight-bearing as tolerated to the right lower extremity and transitioned out of the right arm posterior splint after 1 month, advancing to weight-bearing as tolerated by 2 months. At her 14-week postoperative visit, she was doing remarkably well, ambulating without any pain, with complete union of the right ulna pathologic fracture and reconstitution and ossification of the right proximal humeral and femoral infiltrative lesions (Fig. 7). Of note, this striking response occurred despite having never received radiation, steroids, non-steroidal anti-inflammatories (NSAIDS), or long-term antibiotics (other than routine perioperative prophylactic antibiotics). She was seen again at 9 months postoperatively and had returned to a full active lifestyle without any pain, even reportedly running, per the patient. X-rays showed further interval ossification of the previously infiltrative regions of the bone, without any signs of local recurrence (Fig. 8).

## Discussion

CRMO is a severe form of chronic nonbacterial osteomyelitis (CNO). CRMO and CNO are both autoinflammatory bone disorders; however, CRMO is multifocal and characterized by multiple recurrences leading to a greater impact on a patient’s quality of life [8]. The presence of CNO in a patient indicates a patient may have bone inflammatory disorders like CRMO, SAPHO (synovitis, acne, pustulosis, hyperostosis, and osteitis) syndrome, pustulotic arthro-osteitis, chronic sclerosing osteomyelitis, or lymphoplasmacellular osteomyelitis [9]. CRMO and SAPHO syndrome have been shown to share many of the same characteristics, including osteitis, a multifocal presentation, and hyperostosis and thus are sometimes diagnosed interchangeably. However, they differ in that CRMO is seen mainly in children, affects the extremities and usually lacks skin lesions, whereas SAPHO is seen in adults, affects the axial skeleton, and is characterized by inflammatory skin conditions like acne and pustulosis [2, 10]. CRMO and SAPHO have also been shown to present with characteristically different inflammatory markers [11]. Some believe CRMO is the pediatric form of SAPHO; however, in adult patients with multifocal bone lesions of the extremities without skin lesions, distinguishing between SAPHO and adult CRMO is difficult [2]. Despite their relatively older age, our patient was diagnosed with CRMO, as they presented with multifocal lesions of their extremities without any evidence of cutaneous lesions or hyperostosis. CRMO is a rare disease that has no prior reported cases over the age of 75 making this case the first reported incidence of CRMO in a patient over 75.

CRMO patients typically present with a combination of non-specific musculoskeletal pain, tenderness, swelling, and limited range of motion. This pain may or may not be accompanied by a fever [12]. CRMO is characteristically associated with extra-articular disorders such as palmoplantar pustulosis, psoriasis, Crohn’s disease, acne, and Sweet Syndrome [3]. Upon full body imaging, these patients typically discover bone lesions, myositis, and cutaneous manifestations in asymptomatic areas [2, 13]. Lab findings characteristically show elevated white blood cell counts, and nonspecific inflammatory markers including an elevated erythrocyte sedimentation rate and elevated c-reactive protein levels.

The diagnosis of CRMO is typically a diagnosis of exclusion and thus requires significant imaging and diagnostic studies to rule out other diagnoses. In the current case, plain radiographs, computed tomography (CT), magnetic resonance imaging (MRI), and a skeletal survey were all obtained. Diagnostic imaging alone was not sufficient to establish the final diagnosis; therefore, bone biopsies were performed for further analysis. Bone biopsies are invasive but, in the author’s opinion, were necessary to establish a definitive diagnosis to guide appropriate further treatment. Treating an incorrectly presumed malignancy with chemotherapy and radiation, for example, would recklessly cause unnecessary harm to this patient. This contrasts with Jansson et al. who advocate for establishing a diagnosis based on a scoring system, without a need for biopsy.

The Jansson clinical score specifically ranks chronic nonbacterial osteomyelitis types in an effort to try to avoid requiring bone biopsies to diagnose CNO when patients present with bone pain and inflammation [3, 9]. The score is composed by assigning points based on the number, location, symmetry, presence of marginal sclerosis, temperature, blood cell count, and CRP level. They argue that this scoring system allows physicians to diagnose patients when their clinical history, examination, labs, and radiology findings are indicative of CNO. The presence of CNO can then narrow the differential diagnosis as CNO is a standard for bone inflammatory disorders like CRMO, SAPHO, pustulotic arthro-osteitis, chronic sclerosing osteomyelitis, and lymphoplasmacellular osteomyelitis [9]. Per their study results, Jansson scores >39 indicate CNO with a positive predictive value of 97%. Based on this scoring system, our patient would have a score of 45, which would indicate that theoretically, the diagnosis of CNO could have been made without performing a bone biopsy [2, 7]. Still, the Jansson score algorithm is based on a small cohort of patients which may not be reflective of the true general population, and therefore may not be clinically specific or relevant. The Jansson score at best can help save a patient from a simple biopsy, but at worst, can lead to delayed diagnosis and treatment of potential infectious or malignant processes. We believe the Jansson scoring system may therefore be used to augment a clinician’s existing suspicions for CNO but should never be used solely to make the diagnosis. A strong consideration for biopsy should be made for all indeterminate lesions until proven otherwise.

Treatment of adult CRMO patients typically consists of NSAIDs as a first line therapy, and corticosteroids, methotrexate, sulfasalazine, bisphosphonates, anti-TNF-α, and anti-IL-1R agents with some variation dependent on a patient’s specific needs and medical history. Wipff et al. reported efficacy rates of 41% for sulfasalazine, 37.5% for methotrexate, 75% for bisphosphonates, and 89% for anti-TNF-α agents in treating CRMO. These rates, however, are subjective as treatments were deemed “effective” only by the subjective clinical assessment of a patient’s local physician, rather than any objective measured response [3]. The Sato et al. study also showed anti-IL-6 therapy should be considered in patients with muscle inflammation which does not respond to NSAIDs, methotrexate, or corticosteroids as anti-IL-6 agents can reduce symptoms and inflammatory markers. However, in a study analyzing specific treatments for adults with nonbacterial osteomyelitis of the mandible, researchers found that no evidence-based therapies have accomplished complete remission and that current treatments are focused on alleviation of symptoms [14]. Further studies should be focused on achieving a standardized treatment strategy that can help patients with symptom remediation and achieve remission.

## Conclusion

This is the first report of an octogenarian with CRMO to date. Only one other published case reported CRMO in a patient with a similar age at 74-years-old [7]. In addition, this case is well documented with exhaustive imaging, laboratory testing, and bone biopsy histologic findings to rule out all other possible diagnoses on the differential. The bone biopsy findings in particular may not be seen in other reports due to the establishment of a diagnosis based on Jansson’s clinical score to diagnose CRMO without biopsy. We, however, believe clinicians should be warried to rely on the Jansson score alone to establish a diagnosis, especially for patient outliers such as in this 83-year-old patient. Accurately diagnosing this condition is critical to a patient’s outcome, as overtreatment can lead to major unnecessary comorbidities. Mistakenly radiating this bone due to an assumption of multiple myeloma, for example, or multiple “washout” procedures with prolonged courses of antibiotics for presumed osteomyelitis, would both prove fruitless, with potentially irreversible and harmful final consequences in the setting of CRMO.

Currently, the prevalence of CRMO is probably underestimated due to its status as a diagnosis of exclusion; therefore, more geriatric patients suffering with undiagnosed CRMO are likely to exist [12, 13]. Further research is needed regarding the diagnosis and management of CRMO and should include exploration of the use of biologic agents that target specific cytokines involved in the inflammatory response. This patient’s miraculous recovery despite not receiving any targeted therapy illustrates how little is known about the unpredictable natural history of CRMO. While rare, CRMO should remain in the differential when evaluating adult patients with multifocal bone lesions. Additional investigations could help better understand the pathophysiology of CRMO in the adult and geriatric population, and whether treatment should be different from the pediatric CRMO countertype.

## Data Availability

Readers can access the data used in this manuscript by viewing the supporting radiographic and histologic images which are included in this study. There are no significant statistical data involved in this study.
